# Polyamines and abiotic stress in plants: a complex relationship^1^

**DOI:** 10.3389/fpls.2014.00175

**Published:** 2014-05-05

**Authors:** Rakesh Minocha, Rajtilak Majumdar, Subhash C. Minocha

**Affiliations:** ^1^US Forest Service, Northern Research StationDurham, NH, USA; ^2^U.S. Department of Agriculture, Agricultural Research ServiceGeneva, NY, USA; ^3^Department of Biological Sciences, University of New HampshireDurham, NH, USA

**Keywords:** arginine, biochemical markers, gamma-aminobutyric acid, glutamate, ornithine, proline, reactive oxygen species, stress priming

## Abstract

The physiological relationship between abiotic stress in plants and polyamines was reported more than 40 years ago. Ever since there has been a debate as to whether increased polyamines protect plants against abiotic stress (e.g., due to their ability to deal with oxidative radicals) or cause damage to them (perhaps due to hydrogen peroxide produced by their catabolism). The observation that cellular polyamines are typically elevated in plants under both short-term as well as long-term abiotic stress conditions is consistent with the possibility of their dual effects, i.e., being protectors from as well as perpetrators of stress damage to the cells. The observed increase in tolerance of plants to abiotic stress when their cellular contents are elevated by either exogenous treatment with polyamines or through genetic engineering with genes encoding polyamine biosynthetic enzymes is indicative of a protective role for them. However, through their catabolic production of hydrogen peroxide and acrolein, both strong oxidizers, they can potentially be the cause of cellular harm during stress. In fact, somewhat enigmatic but strong positive relationship between abiotic stress and foliar polyamines has been proposed as a potential biochemical marker of persistent environmental stress in forest trees in which phenotypic symptoms of stress are not yet visible. Such markers may help forewarn forest managers to undertake amelioration strategies before the appearance of visual symptoms of stress and damage at which stage it is often too late for implementing strategies for stress remediation and reversal of damage. This review provides a comprehensive and critical evaluation of the published literature on interactions between abiotic stress and polyamines in plants, and examines the experimental strategies used to understand the functional significance of this relationship with the aim of improving plant productivity, especially under conditions of abiotic stress.

## Introduction

Polyamines (PAs) are small, positively charged, organic molecules that are ubiquitous in all living organisms. The three common PAs in plants are putrescine (Put), spermidine (Spd) and Spm, with some plants also having thermospermine (tSpm) in place of or in addition to Spm. The simplicity of their structure, their universal distribution in all cellular compartments, and presumed involvement in physiological activities ranging from structural stabilization of key macromolecules to cellular membranes make them an attractive group of metabolites to assign a multitude of biological functions. Their accumulation in large amounts in the cell could presumably sequester extra nitrogen (N) thus reducing ammonia toxicity and also balance the total N distribution into multiple pathways. It is not surprising that fluctuations in their cellular contents are often related to varied responses of plants to different forms of stress and to different phases of growth activity. As much as their cellular functions are diverse, and sometimes contradictory, so are their roles in plant stress. They have been deemed important in preparing the plant for stress tolerance and to directly aid in ameliorating the causes of stress, and at the same time, their own catabolic products are responsible for causing stress damage. Several aspects of the relationship between PAs and abiotic stress in plants and their seemingly contradictory roles in the process have been reviewed over the years (Galston and Sawhney, 1990; Alcázar et al., 2006a, 2010, 2011a; Kusano et al., 2007; Liu et al., 2007; Bachrach, 2010; Alet et al., 2011; Hussain et al., 2011; Shi and Chan, 2014).

## Abiotic stress in plants—assessment of the situation

Before delving into the specific roles of PAs in plant stress responses, a few details are important to consider regarding the phenomenon of “abiotic stress.” The first and the foremost is the lack of a precise definition of this term. Each plant constantly faces a changing microenvironment from the moment it starts its growth, be it from a seed or a vegetative cutting. On a daily basis, these changes occur from sunrise to sunset (e.g., light, temperature, changes in CO_2_ and O_2_), and with every cell division, cell enlargement and differentiation activity within the organism. Over its lifetime, there are significant changes in the growth environment; some caused by weather events (like rain or drought), and others part of seasonal changes in temperature and day length. For perennials, there still are the longer-term climatic changes that are relevant to their life. Despite difficulties of precisely defining stress, thousands of experimental studies have involved a variety of stress treatments (mostly short term, i.e., minutes to hours and days) and analysis of the physiological, biochemical and molecular responses of plants to such treatments when they were growing under otherwise “normal” conditions—thus in most cases significant deviation from status quo may be considered stressful.

It is well known that a particular environmental change may be stressful for one species but not for another living under the same conditions. In fact, even within the same species differences exist for response to the same climatic conditions because of genotypic differences among individuals and/or variations in the soil microclimate. A plant's response(s) may involve avoidance of the imposed stress or short-term adaptation to it with the ability to revert back to the original growth and metabolic state. This is in contrast to the evolutionary adaptation (e.g., halophytes, xerophytes, thermophiles) and the long-term physiological adaptations, e.g., those in shade loving plants vs. those that grow better in full sun, and plants requiring large quantities of fertilizer vs. those that can thrive on marginal lands. In most cases the genetics and physiology of a plant allow it to live in a wide range of environmental conditions (as defined by the climate) while in others the range of acceptable environments may be rather narrow. The developmental stage of the plant also plays a significant role in its response to changing environment.

**Figure 1:**
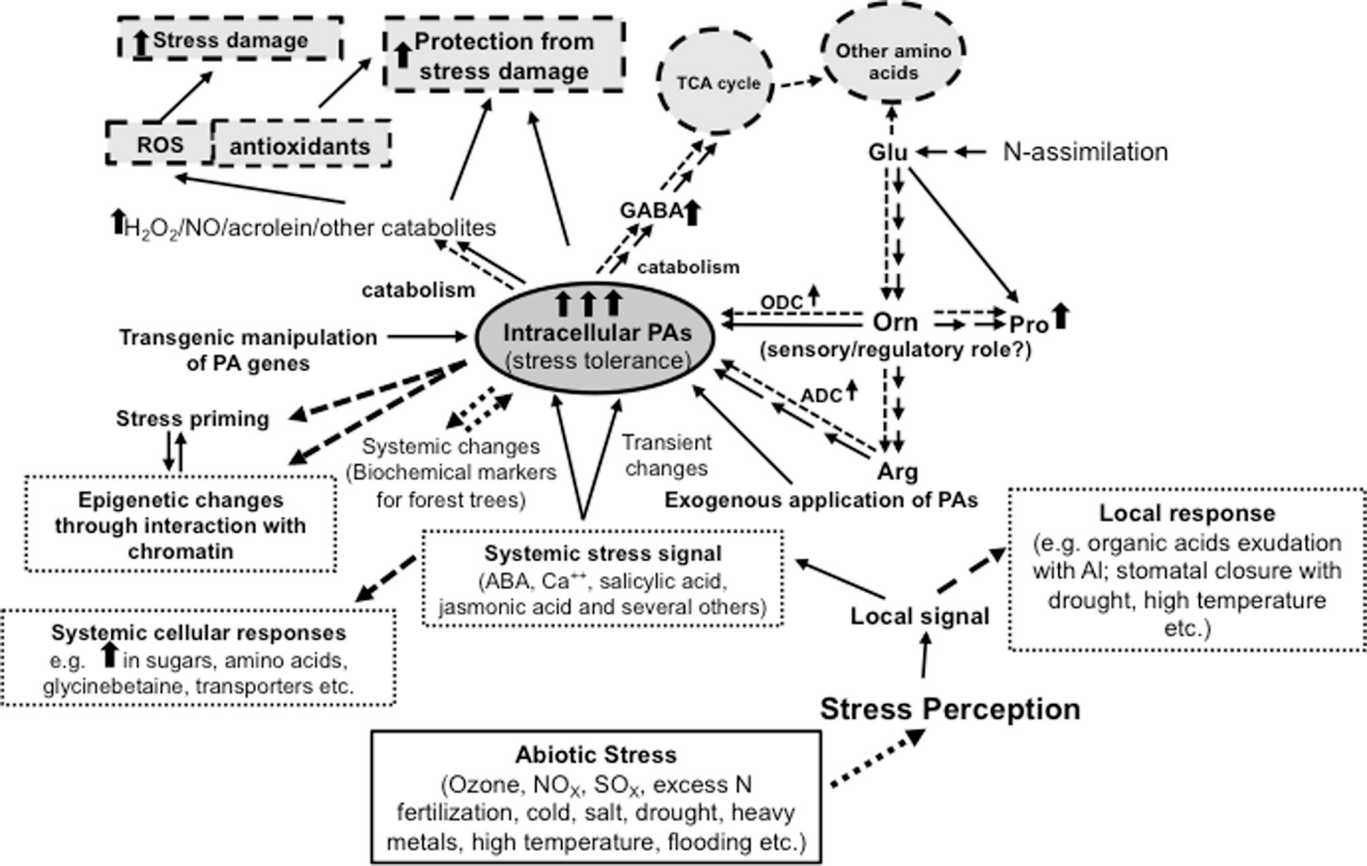
**Diagrammatic representation of the complexity of interactions between polyamines and abiotic stress response in plants**. The Figure also shows a central role of ornithine in the metabolic interaction of polyamines with glutamate, proline, arginine and γ-aminobutyric acid. While multiple arrows indicate multiple steps, the dotted arrows indicate increased flux/positive regulatory role. Thick upright arrows indicate increase in concentrations or effect.

Abiotic stress exposure in plants can be divided into three arbitrary stages: stress perception, stress response and stress outcome (Figure 1). Depending on the nature of stress, its perception can be localized to a specific group of cells, tissues and organs or it could be widespread. Additionally, stress could arise suddenly or slowly. For example, exposure of roots to a heavy metal in fertilizer or saline water or to flooding is likely to be different from that if the plant started its life in the presence of these stressors. On the other hand, drought due to lack of programmed irrigation and/or excessive transpiration, or a gradual increase in ozone concentration in the air, are examples of slow exposure to stress. In the latter instances, the precise organ or tissue perceiving stress is difficult to determine. Therefore, the perception of sudden vs. gradual exposure to stressors can be physiologically quite different and must involve different sensing mechanisms. Likewise, whilst the initial exposure to stress may be limited to a certain plant organ (e.g., roots in the case of salt or heavy metal), yet the response is often systemic. In cases when the tolerance mechanism includes stress avoidance (e.g., exclusion of toxic or harmful chemicals including heavy metals) by interfering with uptake mechanisms, the response is generally limited to the same tissues and/or organs that perceive the stress signal. Yet again, even when the responding tissues are the same as the perceiving tissues, e.g., secretion of organic acids in the presence of Al (Kochian et al., 2004; Yu et al., 2012), the metabolism of the entire organ/plant may be affected with broad tissue-specificity. Hence, explanation of the effects of stress on plant metabolic changes like those in PAs must take into account the experimental conditions being used.

The transmission of the stress signal also involves a multitude of mechanisms; some of which are common for different types of stress. For example, drought, flooding, salt, heavy metals, ozone, and sometimes heat or cold all show a common set of physiological responses, which involve regulatory metabolites like abscisic acid (ABA), salicylic acid and jasmonate or methyl jasmonate (MeJa). Frequently, these modulators of stress may affect metabolites that are common for tolerance and/or amelioration of a variety of stresses (e.g., γ-aminobutyric acid - GABA, proline - Pro, glycinebetaine) or they may be specialized (e.g., phytochelatins in response to heavy metals). Polyamines, in combination with Pro and GABA belong to the former group with almost universal involvement in a variety of stress responses.

## Polyamines and abiotic stress in plants

The history of PAs and their roles in stress tolerance in plants goes back to almost four decades (Hoffman and Samish, 1971; Murty et al., 1971). The issues related to PA functions in stress are especially difficult to study because of their ubiquitous presence and absolute necessity for cell survival, and their presence in relatively large (millimolar) quantities. One of the most confounding problems relating to the role of PAs in abiotic stress is the lack of our understanding of the mechanisms underlying their function(s). The above arguments are consistent with the recent portrayal of PAs by Hussain et al. (2011) as “mysterious modulator of stress response in plants,” perhaps because their roles span a large spectrum of cellular activities but their mechanisms of action are rather poorly understood. The authors cite numerous studies in which overall PA metabolism is increased in response to a variety of abiotic stresses - chemical or physical. Several publications (Alcázar et al., 2006a; Takahashi and Kakehi, 2010; Alet et al., 2011; Hussain et al., 2011; Gupta et al., 2013; Shi and Chan, 2014) have elegantly summarized the various likely roles of PAs in tolerance and/or amelioration of stress in plants. These include: (i) serving as compatible solutes along with Pro, glycinebetaine and GABA; (ii) interactions with macromolecules like DNA, RNA, transcriptional and translational complexes, and cellular and organellar membranes to stabilize them; (iii) role in directly scavenging oxygen and hydroxyl radicals and promoting the production of antioxidant enzymes and metabolites; (iv) acting as signal molecules in the ABA-regulated stress response pathway and through the production of H_2_O_2_; (v) regulators of several ion channels; and, finally (vi) participation in programmed cell death. To this list can be added their role in metabolic regulation of ammonia toxicity, nitric oxide (NO) production, and balancing organic N metabolism in the cell (Nihlgård, 1985; Moschou et al., 2012; Guo et al., 2014).

The facts that PAs are often present in large quantities and their biosynthesis uses Glu, a key amino acid for N assimilation, as the starting material, it can be envisioned that large changes in their biosynthesis and catabolism (e.g., >5–10-fold) could cause major homeostatic shifts in cellular metabolism. Therefore, under conditions of stress, PAs could perform these functions better when changes in their metabolism are transient and within narrower limits, thus avoiding catastrophic perturbations in the overall cellular homeostasis of C and N (Minocha et al., 2000; Bhatnagar et al., 2001; Bauer et al., 2004; Majumdar et al., 2013). However, in perennial trees exposed to persistent environmental stress from air pollutants and resulting changes in soil chemistry, the altered metabolic homeostasis may stabilize enhanced PA levels in a way that they can be used as biochemical markers of stress (Minocha et al., 2000, 2010) In these situations their role could be more prophylactic in preventing stress damage rather than short-term protection. For more details, see Section Polyamines as Metabolic Markers of Long-Term Environmental Stress in Forest Trees.

There are four types of studies that make a strong case in favor of the importance of PAs in plant stress response (Galston and Sawhney, 1990; Alcázar et al., 2006a, 2010; Kusano et al., 2007; Liu et al., 2007; Bachrach, 2010; Alet et al., 2011; Hussain et al., 2011; Shi and Chan, 2014). These include: (i) up-regulation of PA biosynthesis in plants via transgene expression generally increases their tolerance to a variety of stresses; (ii) increased PA accumulation in plants under stress conditions is accompanied by increase in the activity of PA biosynthetic enzymes and the expression of their genes; (iii) mutants of PA biosynthetic genes generally have less tolerance of abiotic stress; (iv) while exogenous supply of PAs makes the plants tolerant to stress, inhibition of their biosynthesis makes them more prone to stress damage. Some highlights of the recent studies in these areas are summarized here:

### Transgenics and stress tolerance

In reviewing the literature on the improvement of stress tolerance in transgenic plants over-expressing a homologous or a heterologous gene encoding a PA biosynthetic enzyme, a few conclusions stand out (for key points of the major studies and references, see Tables 1, 2):

1. Every one of the PA biosynthetic enzyme genes has been expressed as a transgene in several plant species; in most cases a constitutive promoter controls the transgene expression.
2. Experiments with transgenics have typically involved short-term treatments with stress followed in many cases by removal of the treatment to study recovery from stress. Only in a few cases have the plants been brought to maturity and analyzed for total biomass or yield of the desired product (seeds, fruit, or leaf biomass) or its quality (e.g., nutritional properties).
3. Measurements of stress response have included visual symptoms of water loss or wilting, changes in fresh weight, dry weight, ion release, gene expression, and analysis of enzyme activities and cellular metabolite, etc.
4. Transgenic manipulations of *ADC* or *ODC* in plants have resulted in a significant increase in Put content (typically 3–10-fold) with relatively smaller (<2-fold) changes in Spd and Spm. Transgenic manipulation of *SPDS, SPMS*, and *SAMDC* causes smaller (compared to Put) increase of Spd and Spm contents (~2–3 fold).
5. The fold increase in Put content often varies with the plant species, homologous or heterologous gene sources, nature of the promoter, developmental stage of the plant, and the tissues analyzed.

**Table 1:** **Genetic manipulation of *ODC, ADC*, and *SAMDC* genes and enhanced tolerance to abiotic stress in transgenic plants**.

**Plant species**	**Promoter::Transgene**	**Stress application (short or long term)**	**Increase in enzyme activity**	**Increase in Put**	**Increase in Spd**	**Increase in Spm**	**Outcome**	**Citation**
*Nicotiana tabacum* var. xanthi	35S::Mouse *ODC*	NaCl (200 mM; up to 4 week from germination or 15 day old seedlings subjected to 300 mM NaCl for 4 week)	Very high (mouse ODC; native ODC or ADC activity was lower in the transgenics)	2–3-fold	2–3-fold	NS	Greater tolerance to salt stress	Kumria and Rajam, 2002
*Oryza sativa*	ABA-inducible:: *Avena sativa ADC*	NaCl (150 mM; 2-day in 10-day old seedlings)	3–4-fold	1.7–2.2-fold	NA	NA	Increased tolerance to salinity stress	Roy and Wu, 2001
*Oryza sativa*	*35S::Datura stramonium ADC*	Drought (60-day old plants; 6 day in 20% PEG followed re-watering for 3 day)	NA	1.5–4-fold	NS	NS	High tolerance to drought	Capell et al., 2004
*Solanum melongena*	*35S::Avena sativa ADC*	Salinity (150–200 mM NaCl; 8–10 day), drought (7.5–10% PEG; 8–10 day), low temperature (6–8°C; 10 day), high temperature (45°C for 3 h), cadmium (0.5–2 mM for 1 month) in 8–10 day old seedlings	3–4-fold (ADC, DAO), ~2-fold (ODC)	3–7-fold	3–5-fold	~2-fold	Enhanced tolerance to multiple stresses	Prabhavathi and Rajam, 2007
*Arabidopsis thaliana*	35S:*:Arabidopsis thaliana ADC2*	Drought (4 week-old plants for 14 day followed by 7 day recovery)	NA	2–12-fold	NS	NS	Increased tolerance to drought	Alcázar et al., 2010
*Arabidopsis thaliana*	35S: *:Arabidopsis thaliana ADC1*	Low temperature [3 week-old plants for ~9 day at 4-(−11)°C followed by a 2 week recovery]	NA	~3–5-fold	NS	~(−) 1.3–1.9-fold	Greater tolerance to low temperature	Tiburcio et al., 2011
*Arabidopsis thaliana*	*pRD29A::Avena sativa ADC*	PEG (11-day seedlings for 13 h), low temperature (3-week old plants for 10 day)	~10–17-fold (low temp)	~3–5-fold (low temp)	NS	NS	Greater resistance to dehydration and low temperature stress	Alet et al., 2011
*Arabidopsis thaliana adc1-1* mutant	35S: *Poncirus trifoliate ADC*	High osmoticum, drought, and low temperature (up to 14-day from germination, 1–18 day in 3–4 week-old plants)	NA	~2-fold	NS	NS	Enhanced resistance to high osmoticum, dehydration, long-term drought, and low temperature stresses	Wang et al., 2011
*Lotus tenuis*	*pRD29A::Avena sativa ADC*	Drought (6–8 week-old plants exposed to soil water potential of −2 MPa)	~2.2-fold (drought)	~3-fold (drought)	NS	NS	Increased tolerance to drought	Espasandin et al., 2014
*Oryza sativa*	*ABA inducible*: *Tritordeum SAMDC*	NaCl (150 mM; 11 day-old seedlings for 2 day)	NA	1.3-fold (salt)	2.4-fold (salt)	2.8-fold (salt)	Enhanced salt tolerance	Roy and Wu, 2002
*Nicotiana tabacum* var. xanthi	*35S::Homo sapiens SAMDC*	NaCl (250 mM), PEG (20%) up to 2 months from sowing	~1.3–5-fold (overall SAMDC), ~2-fold (DAO)	~2.4–2.7-fold	~1.4–2.4-fold	~1.4-fold	Greater tolerance to salt and drought	Waie and Rajam, 2003
*Nicotiana tabacum*	35S::*Dianthus caryophyl/us SAMDC*	Salt (NaCl; 0–400 mM from sowing through 8 week) Low temperature (5 week-old plants for 24 h at O°G)	2-fold	NS	2.1-fold	1.7-fold	Increased tolerance to oxidative, salt, low temperature, and acid stresses	Wi et al., 2006
*Lycopersicon esculentum* Mill.	35S:: *Saccharomyces cerevisiae SAMDC*	High temperature [35 day old plants for 4 day at 38°C/30°C (d/n) followed by 3 day recovery period]	NA	NS	~1.4-fold	~1.4-fold	Higher tolerance to high temperature	Cheng et al., 2009
*Oryza sativa* L. subsp. Japonica cv. EYI105	*Ubi::Datura stramonium SAMDC*	Osmoticum (PEG; 60 day-old plants for 6 day followed by 20 day recovery period)	NA	NS	1.5–2-fold	NS	Greater tolerance to high osmoticum induced drought and better recovery	Peremarti et al., 2009
*Nicotiana tabacum*	35S:: *Malus domestica SAMDC2*	Low temperature (4°C; 5 day-old seedings for 0, 6, 120 h, and 30 day), PEG (20%; 5 day old seedings for 6 h), NACl (150 mM and 250 mM; 15-day old seedings for 48 h, and 60 day)	NA	1.1–1.5-fold	1.2–1.6-fold	1.7–2.2-fold	Enhanced tolerance to low temperature, high osmoticum, and NaCl	Zhao et al., 2010
*Arabidopsis thaliana*	35S:: *Capsicum annuum SAMDC*	Drought (2 week old plants for 6 h or 3 week-old plants for 11 day followed by 3 day recovery)	1.4–1.6-fold (total SAMDC)	NS	~1.8-fold	~1.7-fold	Increased drought tolerance	Wi et al., 2014

Fold increases of PAs in transgenic plants are from the basal level unless otherwise stated (NA, not available; NS, not significant).

**Table 2:** **Genetic manipulation of *aminopropyl transferase* genes and enhanced tolerance to abiotic stress in the transgenic plants**.

**Plant species**	**Promoter::Transgene**	**Stress application (short or long term)**	**Increase in enzyme activity**	**Increase in Put**	**Increase in Spd**	**Increase in Spm**	**Outcome**	**Citation**
*Arabidopsis thaliana*	*35S::Cucurbita ficifofia SF'DS*	Low temperature (25 day-old plants at −5°C for 40 h followed by 5 day recovery), salinity (75 mM NaCl for 45 day *in-vitro*), high osmoticum (250 mM sorbitol for 70 day *in-vitro*), drought (3 week-old plants for 15 day), oxidative stress (leaf discs at 0.5–5 μM for 14 h)	5–6-fold (SPDS)	NS	1.3–2-fold	1.6–1.8-fold	Increased tolerance to low temperature, salinity, hyperosmosis, drought, and paraquat toxicity	Kasukabe et al., 2004
*lpomoea batatus*	*35S::Cucurbita ficifolia SPDS*	Salt (NaCl; 114 day from planting), Low temperature (10–30°C for 6 h), high temperature (42–47°C for 5 min)	NA	1.5-fold	2-fold	NS	Enhanced tolerance to salt, drought, extreme temperatures, and oxidative stress	Kasukabe et al., 2006
*Pyrus communis* L. “Ballad”	*35S::MaIus sylvestris* var. *domestica SPDS*	Salt (250 mM NaCl for 10 day), high osmoticum (300 mM mannitol for 10 day), heavy metals (500 μM CuSO_4_ for 15 day; 30 μM AlCl_3_, 50 μM CdCl_2_, 500 μM PbCl_2_, and 500 μM ZnCl_2_ for 21–30 day *in-vitro*)	NA	1.1–1.6-fold	1.3–1.9-fold	0.6–1.7-fold	Greater tolerance to salt, high osmoticum, and heavy metals	Wen et al., 2008, 2009, 2010
*Lycopersicon esculentum*	*35S::MaIus sylvestris* var. *domestica SPDS1*	Salt (100 or 250 mM NaCl; 4 week-old plants for 60–65 day)	NA	NS	~1.5–1.6-fold	NS	Enhanced tolerance to salt stress	Neily et al., 2011
*Arabidopsis thaliana*	*35S::Arabidopsis thaliana SPMS*	High temperature (7–15 day-old seedlings for 0.5–2.5 h at 42–45°C)	NA	1.6-fold	(−)4–6-fold	4.4–4.7-fold	Enhanced tolerance to thermal stress	Sagor et al., 2013

Fold increases of PAs in the transgenic plants are above the basal level unless otherwise stated (NA, not available; NS, not significant).

### Enzyme activity and gene expression of polyamine biosynthetic enzymes

An increase in cellular PAs in the initial stages of stress treatment is often accompanied by increased activity of Put biosynthetic enzymes like ADC and ODC, but generally not those involved in the biosynthesis of higher PAs, i.e., SAMDC, SPDS, and SPMS (Majumdar et al., 2013). This observation is consistent with the specificity of response being limited often to changes in Put in most cases, and also that the cellular contents of higher PAs often change only within a narrow range (Minocha et al., 1997, 2000, 2010; Bhatnagar et al., 2002; Majumdar et al., 2013). In some cases, where two or more copies of a gene encoding the same enzyme are present, often a general increase in gene expression for all copies is observed (Urano et al., 2004, 2009; Hu et al., 2005; Do et al., 2013; Guo et al., 2014).

The detailed functional enrichment analyses have been reported for differential gene expression in high Put-producing transgenic *A. thaliana* over-expressing a homologous *ADC2* gene using microarrays (Alcázar et al., 2005; Marco et al., 2011a,b). The results showed that the direct target of increased Put accumulation included genes responsive to salt, heavy metals, cold, and oxidative stresses, besides those involved in basic cellular processes, e.g., translation and ribosome structure. Several genes associated with IAA biosynthesis, transport and auxin-related transcription factors, ABA biosynthesis and ABA-related transcription factors, and other signal transduction-related genes were also significantly up regulated in the transgenic plants. On the other hand, Spm over-producing transgenic *A. thaliana* (with a homologous *SAMDC1* or *SPMS* transgene) positively affected defense-related (biotic/abiotic stresses) genes, signaling pathway genes (e.g., mitogen activated protein kinases - MAPKs), and genes associated with ABA-, JA-, and SA- biosynthesis related enzymes. The commonalities of up-regulated stress-related gene expression in Put and Spm-overproducers (e.g., Ca^++^ signaling-related genes and ABA biosynthetic genes) suggest overlapping functions of Put and Spm (by interacting with ABA or modulating Ca^++^ homeostasis), which are common to tolerance against drought, salt or low temperature.

In a comprehensive study of two rice cultivars (*Oryza sativa* L. ssp. *indica* and *japonica*) kept under 18 days of drought stress, Do et al. (2013) noted up-regulation of several genes and related metabolites involved in PA biosynthesis via Orn/Arg pathways. Of the 21 genes associated with the Orn/Arg pathway, 11 co-localized with the drought-related QTL regions. Although Put was the predominant PA under normal conditions, Spm became dominant upon exposure to drought indicating Put to Spm conversion, similar to what Alcázar et al. (2011b) had observed in *A. thaliana*. There was also an increase in the expression of selected paralogs of *SAMDC, SPDS*, and *SPMS* genes. However, in a comparison of high and low Put cell lines of *Populus nigra x maximowiczii* growing in suspension cultures, Page et al. (2007) found no major differences in the expression of most of the genes (q-PCR) of the Glu-Orn-Arg/Pro/Put pathway, indicating that increased flux of Glu→Orn (in high Put cells) was not transcriptionally regulated.

Recently the role of endogenously produced Put affecting the expression of drought responsive gene 9-cis-epoxycarotenoid dioxygenase (NCED) - can enzyme that controls ABA biosynthesis under stress, was studied in lotus (*Lotus tenuis*), using a heterologous oat (*Avena sativa*) *ADC* gene under the control of a drought/ABA inducible promoter *RD29A* (Espasandin et al., 2014). Drought increased the expression of oat *ADC* by ~100-fold, total ADC activity by ~2-fold and Put by ~3.6-fold, with only minor changes in Spd and Spm. The non-transgenic plants showed relatively smaller changes of these parameters upon exposure to drought. Higher Put contents in the transgenic plants significantly increased (~3-fold) the expression of *NCED* as compared to the wild type plants, suggesting the possibility of transcriptional regulation of ABA synthesis by Put.

### Mutants of polyamine biosynthetic genes and stress

Mutants for almost all of the key PA biosynthetic genes have been tested for their stress tolerance properties. Since PAs are an absolute requirement for growth in all organisms, and most PA biosynthetic genes are present in at least two copies in plants, knockouts for single gene mutants are often the only feasible approach to study their involvement in stress. Based on extensive analysis of Arabidopsis mutants, no single gene of the pathway has been found to be absolutely essential or to play a specific role in stress response. The mutant studies further showed a reduction in seed development in cases where more than one gene was affected (Imai et al., 2004; Urano et al., 2005; Ge et al., 2006), hence maintenance of homozygous double mutants of the two gene copies encoding the same enzyme has not been possible.

An Arabidopsis double knockout (*acl5/spms*) compromised for tSpm and Spm biosynthesis showed hypersensitivity to NaCl and KCl but not to MgCl_2_ (Yamaguchi et al., 2006; Alet et al., 2011). Altered tSpm and Spm levels in the mutant were shown to impair Ca^++^ homeostasis thereby affecting their overall monovalent: bivalent charge ratio leading to a differential response to salts. In addition to salt and drought stresses, Spm also plays a significant role in heat tolerance as shown by hypersensitivity of a T-DNA insertion mutant of *SPMS* in Arabidopsis exposed to higher temperature. The hypersensitivity was overcome either by exogenous supply of Spm (and tSpm) or by increasing endogenous Spm by constitutive over-expression of a homologous *SPMS* (Sagor et al., 2013).

An Arabidopsis single mutant of *ADC* (*adc1* or *adc2*) with significantly reduced Put content showed increased sensitivity to low temperature (Cuevas et al., 2008). The *adc* mutants showed reduced expression of *NCED3* (ABA synthesis). Complementation and reciprocal complementation with ABA and Put, respectively improved low temperature tolerance of the mutants.

A potential role of ABA (Christmann et al., 2005) in the induction of genes that encode PA biosynthetic enzymes was demonstrated independently (Urano et al., 2004, 2009; Alcázar et al., 2006b). This interaction was further confirmed by studies with ABA-deficient mutants (in which increase in ADC under stress was not seen), analysis of the promoter regions of several PA biosynthetic enzyme genes that have ABRE-like motifs, and the direct effects of applied ABA on Put production.

### Abiotic stress and exogenous supply of polyamines

Besides transgenic up-regulation of cellular PAs, exogenous application of PAs also shows similar results, i.e., increased stress tolerance, while chemical inhibition of their biosynthesis exhibits increase in damage from stress. Protection from exogenous PAs could presumably come from their direct interactions with the membranes, reduction of oxidant activity, serving as compatible osmolites or their ionic properties (Hu et al., 2005; Ndayiragije and Lutts, 2006; Wang et al., 2007; Afzal et al., 2009). With respect to the use of exogenous PAs and/or the inhibitors of PA biosynthesis, most studies have been restricted to *in vitro* use of callus, leaf explants or young plants, and, with a few exceptions, that have involved short-term stress treatments. The following recent reports are examples of the effects of exogenously supplied PAs on stress response in plants:

➢ Foliar spray of 0.1 mM Put in wheat (*Triticum aestivum* L.) at the time of anthesis and prior to application of drought stress, significantly increased photosynthetic attributes, increased contents of Pro, total amino acids and soluble sugars, improved water status, reduced membrane damage, and significantly increased total grain yield as compared to the control plants (Gupta et al., 2012).
➢ Using an *in vitro* system of detached tobacco leaf discs, Kotakis et al. (2014) found that pre-treatment with 1 mM Put 1 h prior to polyethylene glycol (25%) induced osmotic stress, prevented significant water loss and maintained maximum photosystem II photochemical efficiency (*F*_v_/*F*_m_).
➢ Foliar application of 2.5 mM Put or Arg increased tolerance to high temperature (35 ± 2°C for 4–8 h) in 30–35 day old wheat (*T. aestivum* cv. Giza 168) plants. At 5 days after spray, the plants had higher amounts of endogenous Put, Spd and total amino acids, and lower amounts of ammonium and ethylene. Total yield at 155 days in the Put-treated plants was higher vs. the controls (Hassanein et al., 2013).
➢ Exogenous application of Spd (for 7 days) at early booting stage of rice (*Oryza sativa* L. ssp. *indica*) prior to treatment with NaCl (that continued till maturity) significantly increased grain yield, Ca^++^ content in the grains, and a higher K^+^/Na^+^ ratio as compared to the non-treated control plants (Saleethong et al., 2013).
➢ Exogenous Spd added at the same time as NaCl increased cellular contents of Spd, Spm and Pro in *Panax ginseng* seedlings by activating antioxidant-based defense system, thereby reducing H_2_O_2_ and superoxide molecules (Parvin et al., 2014).
➢ Similar effects of exogenous PAs on ameliorating NaCl stress in 5-month old sour orange (*Citrus aurantium* L.) plants were seen by Tanou et al. (2014). They suggested that it was due to reprogramming the oxidative status of cells by increased expression of genes producing antioxidant enzymes. Proteomic studies reveal reduced protein carbonylation and tyrosine nitration, and increased protein *S*-nitrosylation by PAs.
➢ In a detailed study with Bermuda grass (*Cynodon dactylon*) Shi et al. (2013) found that exogenous PAs, while mitigating drought and salt stresses, significantly increased the abundance of antioxidant enzymes and several other stress-related proteins. These results are consistent with what Zhao et al. (2010) had earlier reported in the same species, where water deficit significantly affected proteins associated with photosynthesis and antioxidant-mediated defense pathways. These results reinforce the role of intracellular Put positively affecting photosynthetic machinery with enhanced capabilities of transgenic plants for biomass accumulation reviewed by Sobieszczuk-Nowicka and Legocka (2014).

## Reactive oxygen species and polyamine catabolism

A multifaceted interaction of PAs with Reactive Oxygen Species (ROS) and antioxidants is perhaps among the most complex and apparently contradictory physiological and biochemical interactions in plants. A functional association between ROS and abiotic stress has been known from the time of their discovery since ROS are capable of causing widespread damage to a variety of cellular metabolites as well as macromolecules (Pottosin et al., 2014 and references therein). Some of the overlapping responses of plants to drought, salinity and other abiotic stresses are presumably related to maintaining a healthy water status in the cells, which requires the removal of ROS and related free radicals involving oxygen. Thus, increase in ROS production in stress tolerant plants is often accompanied by increased biosynthesis of antioxidants and associated antioxidant enzymes to ameliorate the ROS from cellular environment.

Numerous studies have emphasized the complexity of interaction between PAs and the ROS, especially when plants are under stress (Bhattacharjee, 2005; Gill and Tuteja, 2010; Velarde-Buendía et al., 2012; Pottosin et al., 2014). Typically when cellular PA contents are up, their catabolism also increases, the levels of H_2_O_2_ increase, and various ROS as well as the antioxidant systems (enzymes and metabolites) are also up, hence their roles in preventing damage from stress are beneficial as well as deleterious. The role of PAs in augmenting antioxidant based defense systems to impart tolerance against heavy metals, UV and other stresses that are potent inducers of superoxide molecules causing oxidative damage to the living cells have been reported in several studies (Bouchereau et al., 1999; Groppa et al., 2007; Thangavel et al., 2007; Mapelli et al., 2008; Rakitin et al., 2009; Jantaro et al., 2011; Pothipongsa et al., 2012; Chmielowska-Bąk et al., 2013; Mandal et al., 2013; Scoccianti et al., 2013). This is consistent with the diverse roles of PAs including the fact that an increase in cellular PA titers contributes to both sides of the ROS-antioxidant equation under conditions of stress. While on one side the ROS participate in abiotic (and biotic) stresses as parts of signal transduction pathways to induce protective responses (Moschou et al., 2012), on the other side they also directly cause membrane damage, chlorophyll destruction and oxidation of several important metabolites in the cell. Likewise PAs have been implicated in several protective responses in the cell (Bouchereau et al., 1999; Zepeda-Jazo et al., 2011; Pothipongsa et al., 2012; Tanou et al., 2012, 2014), including the protection of membranes and other macromolecules, which are the targets of ROS damage. Furthermore, PA catabolism can contribute directly to cell damage, interestingly via the production of H_2_O_2_ and acrolein as observed in tobacco cells (Kakehi et al., 2008; Mano, 2012; Takano et al., 2012) as well as mammalian systems (Sakata et al., 2003; Yoda et al., 2006; Mohapatra et al., 2009; Saiki et al., 2009; Yoshida et al., 2009). Yet the same source of H_2_O_2_ (i.e., PA catabolism) is needed for lignin production in the apoplast adjacent to the plasma membrane (Moschou et al., 2008).

A major interaction between PAs and ROS presumably occurs at the level of plasma membrane where PAs (due to their strong positive charge) can effectively block cation channels (Williams, 1997; Dobrovinskaya et al., 1999a,b; Zepeda-Jazo et al., 2008; Bose et al., 2011; Zepeda-Jazo et al., 2011). Their specificity for selectively blocking outward Na^+^ channels (vs. the K^+^ channels) in the tonoplast membrane apparently helps the vacuole to contain Na^+^ within it, thus changing the effective K^+^/Na^+^ ratio in the cytoplasm under conditions of stress. Other interactions of PAs with the ion channels have been discussed in several recent reviews (Del Rio and Puppo, 2009; Demidchik and Maathuis, 2010; Pottosin et al., 2012, 2014).

## Polyamines and stress memory/priming

Bruce et al. (2007) have elegantly described the importance of evolutionary and long-term adaptation to environmental stress in plants. They postulate that plants' responses to short-term exposure to stress are governed by a combination of their innate ability (genetic and evolutionary) as well as previous events in the life of the individual plant, i.e., exposure to stress during early development, which they term as “priming.” While the evolutionary basis of adaptation to various abiotic and biotic stresses is obvious from the distribution of plants in different macro- and micro-ecosystems, there are numerous examples of the effects of priming on physiological responses of plants to repeated exposure to several different types of stresses (Jisha et al., 2013 and references therein). These include tolerance to salt, transient exposure to drought, flooding, high and low temperature, and ozone. In other cases of growth under conditions of steady stress (e.g., salt or heavy metals in the soil), priming usually occurs during seed germination, which may have longer-lasting effects on the growth of a plant, even though the overall growth rate may be slower. Adaptations of different species or different ecotypes of the same species to different environments are excellent examples of a combined role of priming and genetic selection within a continuum of climatic/environmental conditions. It was suggested that priming due to early exposure of plants to various forms of abiotic stress might involve epigenetic changes that are stable over the life of a plant.

Epigenetic effects of the changing environmental on gene expression are widely accepted; however, the mechanism of such epigenetic adaptations is not well understood. It is now known that epigenetic changes mostly occur at the level of chromatin, and involve sequence-specific DNA methylation, histone acetylation and methylation, and other similar modifications. While most of the epigenetic changes are stable within the life of an organism, others are reversible through exposure to certain growth and development regulators, and still others appear to be transmitted to the next generation through sexual reproduction (Sano, 2010; Shao et al., 2014; Sharma, 2014). Control of totipotency by plant hormones in cell and tissue culture and stem cell research in animals are excellent additional examples of the role of various external chemical and physical factors in controlling epigenetic changes that regulate cell fate.

As discussed above in relation to the cellular functions of PAs, one could envision a critical role for them in affecting epigenetic changes related to priming for stress. It can be argued that increased PA accumulation in response to short-term stress affects the epigenetic modifications of DNA and histones because of their ability to interact with chromatin (Pasini et al., 2014 and references therein). Important aspects of this premise would include: (i) a fundamental role for PAs in epigenetic changes that normally occur in the life of an organism through specific interactions with DNA prior to or during methylation (Krichevsky et al., 2007; Sharma et al., 2012), and (ii) the enhancement of specific epigenetic changes occurring under conditions of priming for stress (cold or salt treatment of seeds during germination to develop tolerance, desiccation of seeds and buds during dormancy, etc.).

## Polyamines, proline, nitric oxide, arg and γ-aminobutyric acid—the ornithine connection

It is interesting to note that changes in cellular contents of PAs and Pro often seem to occur in a coordinated manner rather than the two moving in opposite directions even though their biosynthesis shares a common precursor, i.e., Glu (Delauney and Verma, 1993; Aziz et al., 1998; Mattioli et al., 2009; Mohapatra et al., 2010; Verslues and Sharma, 2010). When PA biosynthesis is increased - either in response to abiotic stress or through genetic manipulation of ODC or ADC - (Wen et al., 2008; Majumdar et al., 2013), it obviously must cause an increase in the flux of Glu into Orn and Arg, depending upon the route of Put biosynthesis (ODC or ADC). In this regard, we (Page et al., 2007) have shown that the increased flux of Glu to Orn and Arg is apparently regulated at the biochemical level without involving major changes in the expression of genes encoding various enzymes of this pathway. Also, under both situations Pro content increases. However, it is not always clear as to what pathway is involved in increased Pro biosynthesis, i.e., directly from Glu by Δ^1^-pyrroline-5-carboxylate synthetase (P5CS) or from Orn by Orn aminotransferase (OAT). A third metabolite whose cellular content seems to follow the same pattern as Put and Pro is GABA, which, like Pro, is also synthesized by two alternate pathways—from Glu by Glu decarboxylase (GAD) and from Put by diamine oxidase (DAO). An additional product of the Glu/Pro/Arg/Put/GABA pathway is nitric oxide (NO), whose role in various developmental and physiological processes in plants has recently drawn serious attention (Tun et al., 2006; Mur et al., 2013; Tanou et al., 2014); the production of NO also increases under abiotic stress conditions (Wimalasekera et al., 2011). Consequently, increased flux of Glu through this set of reactions must cause either a major loss of cellular Glu (which is not tolerable if the cells must continue other metabolic functions involving Glu; the minimum being the biosynthesis of proteins and other amino acids) or a coordinated enhancement of its biosynthesis via increased N assimilation (or protein degradation, which could happen under conditions of stress). It can thus be argued that these interactive pathways may involve a common signal and/or a common signaling mechanism (Figure 1). The possibility of Orn being involved in such a monitoring and/or signaling pathway for the biosynthesis of all of these metabolites (i.e., Pro, Put, GABA, and perhaps also Arg and NO - Orn is an intermediate for all) has been suggested (Majumdar et al., 2013). They proposed the existence of a mechanism to monitor cellular Orn, and through as yet unknown signaling pathway, increase the flux of Glu into the said pathway, without concomitant effects on the production of Arg. Of course, in the case of abiotic stress, the role of ABA, salicylic acid, jasmonic acid, and other signaling molecules must also be considered.

Ornithine is a non-protein amino acid that is synthesized from Glu (major metabolic entry point of inorganic N in plants) via several steps (Slocum, 2005). It is a metabolic intermediate rather than a terminal product of the PA-amino acid biosynthetic pathways, and occupies a pivotal position contributing to the production of PAs, Arg, and Pro. Augmentation of intracellular Orn titers by manipulation of genes related to Orn biosynthesis has been shown to increase stress tolerance in plants (Kalamaki et al., 2009a,b). Transgenic Arabidopsis plants constitutively over-expressing a tomato *N*-acetyl-L-Glu synthase gene (*SlNAGS1*) showed up to 9-fold increase in foliar Orn, which was accompanied by a small but significant increase (10–29%) in citrulline and a decrease (~20%) in Arg levels. Transgenic lines showed increased germination % and higher root tolerance index when grown in media containing 250 mM NaCl; there also was greater tolerance to salt or drought in mature plants as indicated by their bigger leaf size, and higher growth and chlorophyll contents under stress situations. Additionally, the transgenic plants showed better recovery after stress withdrawal.

Besides increasing intracellular Orn titers through genetic manipulation, exogenous application of Orn has also been shown to alleviate abiotic stresses (Ghahremani et al., 2014). In tobacco cells subjected to NaCl stress, application of exogenous Orn significantly increased the activity of antioxidant enzymes, e.g., catalase (325%), peroxidase (270%) and superoxide dismutase (374%), concomitant with significant increases in Put/Spd and significant decrease in H_2_O_2_ vs. the control cells. Similar stress ameliorating properties of Orn were observed in tobacco cells exposed to high osmoticum (polyethylene glycol); interestingly though, a differential role of D-Orn and L-Orn was observed where the former was more effective under salinity and the latter under drought conditions (Ghahremani et al., 2014).

Correlation between cellular reserves of Orn either as physiological responses of plants to seasonal changes or exogenous application of Orn and tolerance to extreme conditions are also evident from other studies. In leafy spurge (*Euphorbia esula* L.), a perennial weed, a significant increase in free amino acids and soluble protein were observed as an overwintering strategy (Cyr and Bewley, 1989). Cellular Orn increased by 6- to 8-fold in the roots during peak winter compared to the summer months. In detached leaves of cashew (*Anacardium occidentale* L.) plants, exogenous Orn (but not Glu) along with salt stress showed a 2–3-fold increase in Pro contents, suggesting Orn as an effective precursor for Pro accumulation (Da Rocha et al., 2012).

## Polyamines as metabolic markers of long-term environmental stress in forest trees

Whereas the topics of correlations between changes in PAs and the response of plants to short term applications of stress have received generous treatment in the literature, the feasibility of using PAs as potential metabolic markers/indicators of environmental stress in plants before the appearance of visual symptoms (e.g., easily measurable growth effects) has received only limited attention. This application is quite relevant to monitoring the health status of perennials in commercial plantations as well as in managed and unmanaged natural forests. Several studies on analysis of foliar metabolites and soluble inorganic ions in mature forest trees have shown a strong positive correlation between PAs (particularly Put) and chronic effects of environmental stress from acid precipitation or excessive N fertilization of soils. The results suggest a potential for developing guidelines to include such biochemical analysis in forest management practices for stress amelioration.

### Need for monitoring the impact of environmental stress on forest trees

Oxides of sulfur (S) and N, emitted into the environment from industrial processes (e.g., fuel combustion and transportation) react with water to form strong inorganic acids, which make up the major component of acidic deposition (*a.k.a.* “acid rain”). These acids solubilize Ca^++^ from its bound form in the soil, enabling the plant to absorb it easily, thus initially the increase in mobile Ca^++^ may help trees to grow better. However, the acidity also mobilizes aluminum (Al), which does two things: (i) it blocks the uptake of Ca^++^ by roots and (ii) it binds very tightly to soil particles, thereby displacing Ca^+^ from the soil, which eventually leaches from the watershed to surface water bodies (e. g. lakes). The net result is a serious accumulation of soluble N in the inland water bodies (from high N inputs directly from acid rain as well as from the agricultural land and forest runoffs), thus causing algal blooms and harm to other biota. Consequently, some forest soils have become depleted of Ca^++^ to the point where select tree species have developed Ca^++^ deficiency; this has happened in the US (Lawrence et al., 1995; Bailey et al., 1996; Likens et al., 1998; Kobe et al., 2002; Huntington, 2005), Europe (Thimonier et al., 2000; Jönsson et al., 2003) and Asia (Nykvist, 2000). At the same time, N deposition has also reached a level that has either caused or will cause significant harm to the functions and structure of forests (Van Breemen and Van Dijk, 1988; Nykvist, 2000; Galloway et al., 2004; Pardo et al., 2011). The phenomenon of environmental N deposition exceeding its biological demand in forested watersheds is referred to in the literature as “N saturation” (Aber et al., 1989).

Identification of biochemical and physiological markers (e.g., organic metabolites and inorganic ions) in plants whose concentrations change in response to a single or multiple stressor(s) in a predictable (and stable) manner can be useful in monitoring the status of stress response and recovery due to stress ameliorating treatments. Only a handful of metabolic markers linked to specific functions have been identified in plants growing under chronic stress conditions. Phytochelatins are one example of such markers for heavy metal exposure. To be of practical value in assessing community/forest health, the biochemical markers should have the following characteristics: (a) the cost to develop and test them should be reasonable, (b) they should be relevant to ecosystem function that is under study, (c) they should be sensitive and dose responsive so that change in their cellular content should be higher in magnitude relative to “normal” background fluctuations, (d) they should maintain a longer-term new homeostatic level under persistent stress conditions such as those found in forests, (e) their concentration should revert to normal range when the stress inducer is removed from the environment, and (f) they should have broad applicability over temporal and spatial ranges (Gârban et al., 2005); http://www.esd.ornl.gov/programs/bioindicators/typesandcharacteristics.htm; http://www.fda.gov/ohrms/dockets/ac/01/briefing/3798b1_04_holt/sld005.htm. The PAs seem to have many of these characteristics; therefore, we have proposed to use them as biochemical markers of N saturation stress and Ca depletion/Al accumulation in the Northeastern US forests based on analysis of easily accessible foliage.

### Polyamines as biochemical markers for stress

The signs of environmental stress in trees often develop slowly, i.e., in comparison with those from insect or disease damage. For example, visual symptoms of drought stress or nutrient deficiencies may take several years to appear. Unfortunately, once the symptoms of stress and damage become apparent it is often too late to stop or reverse the decline in forest productivity. As mentioned above, it is often difficult to diagnose the source of stress on trees because multiple factors work together (e.g., Al toxicity, N saturation, nutrient deficiencies, ice or wind storm etc.) to cause the decline in forest productivity over a period of many years. Moreover, different tree species have different tolerance limits for each type of stress. Thus, a comprehensive analysis of the growing environment in an ecosystem (soil, water, animal and plant physiology, and above- and below-ground biomass, including microbes) by multidisciplinary teams that include physiologists, ecologists, pathologists, microbiologists, biogeochemists, and hydrologists must be carried out to assess the complete situation. The joint data collected concurrently would help us to develop links between tree function and environmental disturbances, and risk assessment and stress remediation strategies for forest trees prior to the onset of obvious decline. In this regard, based on studies spanning over multiple years in several ecosystems, including some National Science Foundation (NSF) funded Long-Term Ecological Research (LTER) Sites (www.lternet.edu/lter-sites) within US, we have found PAs (especially Put), along with some of the related metabolites (e.g., GABA and Pro) and inorganic ions to be reliable markers of plant health under conditions of environmental stress or soil nutrient deficiency in forest trees. Their contents in plant tissues like foliage in forest trees show strong correlations with a variety of abiotic stress conditions before the damage due to these conditions is visible.

In our attempts to test the utility of these metabolites as biochemical markers for abiotic stress, our studies have involved multiple sites in the Northeastern US that were either chronically impacted by environmental acid precipitation due to NOx and SOx or experimentally fertilized with N (NH_4_NO_3_) to simulate the effects of chronic N addition to the soil (Minocha et al., 1997, 2000; Bauer et al., 2004). Fertilization with Ca was also conducted to study the amelioration of Ca^++^ deficiency symptoms (Wargo et al., 2002; Juice et al., 2006; Minocha et al., 2010). One such study involved six red spruce (*Picea rubens* Sarg.) stands in three NE US states, which had suffered long-term exposure to acidic deposition from industrial sources, and where the soil solution pH in the organic soil horizon (Oa) was <4.0. The presence of a large number of dead and dying trees at some of the sites indicated that they were apparently under some form of environmental stress. Analysis of soil chemistry and PAs in needles from apparently healthy trees over a 2-year period revealed: (i) a strong positive correlation between Ca^++^ and Mg^++^ in the needles and in the Oa horizon of the soil; and (ii) that needles from trees growing on relatively Ca^++^-poor soils with a high exchangeable Al concentration in the soil solution had significantly higher concentrations of Put than those growing on Ca^++^-rich soils with a low exchangeable Al concentration (Minocha et al., 1997). The magnitude of change in Put was several-fold higher than for Spd or Spm. Putrescine concentration in 1 year-old needles always positively correlated with exchangeable Al (*r*^2^ = 0.73, *p* ≤ 0.05) and soil solution Al: Ca^++^ ratios (*r*^2^ = 0.91, *p* ≤ 0.01) of the Oa soil horizon. The study revealed that foliar Put concentration could be used as a reliable biochemical marker for early detection of stress due to soil Ca^++^-deficiency in natural forests before the appearance of any visual symptoms of stress damage.

In another study at the Delaware River Basin NY, USA along a N deposition gradient (from aerial NOx), foliar Put concentration in sugar maple (*Acer saccharum*) increased with the elevation which was accompanied by rise in ambient level of N deposition. Accompanying changes in soil chemistry included decrease in soil Ca^++^ with increasing N (Ross et al., 2004). Additional evidence for the importance of Ca^++^ in controlling foliar Put came from an experimental addition of Ca^++^ to a large watershed (11.7 ha) at Hubbard Brook Experimental Forest NH, USA. In this case, amelioration of soil Ca^++^ improved the overall health of sugar maple trees and increased seedling regeneration, which was accompanied by a concomitant decrease in foliar Put content. The data further revealed that changes in Put were species-specific in that increased Put indicated stress effect only in species that showed Ca^++^ deficiency in regard to the ambient levels of Ca^++^ in the soil. For example, sugar maple, which is known to be more sensitive to Ca^++^ deficiency showed bigger changes in Put relative to yellow birch (*Betula alleghaniensis*) that was less sensitive to existing soil Ca^++^ levels (Minocha et al., 2010). An earlier study at an un-glaciated forest at the Allegheny Plateau, PA, USA aimed at identifying the factors contributing to sugar maple decline involved a one-time treatment with Ca^++^ to a Ca^++^-depleted stand, which also showed improved tree vigor and growth (Wargo et al., 2002). It is surmised that increased foliar Put could possibly substitute for some of the functions of Ca^++^ within the cells as suggested by Minocha et al. (1997).

Long-term (>10 years) studies on chronic N fertilization [NH_4_NO_3_ and (NH_4_)_2_SO_4_] at Harvard Forest MA, USA (HF) and Bear Brook Watershed ME, USA (BBWM), where N fertilization began in 1989, have shown that N-amended pine (*Pinus strobus*) and hardwood (red maple - *Acer rubrum* and red oak - *Quercus rubra*) stands at HF and mixed-wood (red spruce - *Picea rubens*, American beech - *Fagus grandifolia* and sugar maple) stand at BBWM contained higher concentrations of PAs and amino acids in their foliage (Minocha et al., 2000; Bauer et al., 2004) and R. Minocha et al. unpublished data. The changes at both sites were also species-specific and depended on land use history of the sites that had affected their soil N status. There also was a concomitant increase in foliar N from the uptake of ammonia and changes in foliar base elements that were associated with soil base cation losses and nitrate leaching (Aber et al., 2003; Fernandez et al., 2003; Aber and Magill, 2004; Elvir et al., 2005, 2006; Fernandez and Norton, 2010). Data collected over 15 years revealed that N saturation in the pine stand had increased the death of trees at HF. However, in the hardwood stand within half a mile of the pine stand, while maple trees did not survive the chronic N deposition and drought stress of 1998, oak showed no visual symptoms of stress until 2002 (Magill et al., 2000, 2004). Changes in the aboveground tree productivity of both stands was accompanied by changes in the belowground (i.e., soil) fungal and microbial biomass at the HF site (Frey et al., 2004; Wallenstein et al., 2006). In both soil horizons (organic and mineral) at the N-amended hardwood stand, significant rearrangements in bacterial community structure were observed after 20 years of annual N treatment (Turlapati et al., 2013). Biochemical analysis of foliage revealed that Put concentrations were reflective of N storage in most species, and in some, the increase was additive from the response to Ca^++^ deficiency caused by soil nutrient leaching.

In addition to using Put as a biochemical marker of stress, we also found it to be a useful marker to follow the recovery of forest trees from catastrophic stress-causing events like fires, ice damage and silvicultural thinning practices at different sites within the Northeastern and Western regions of the US (Minocha et al., 2013) and R. Minocha et al. unpublished data. Studies were also conducted to demonstrate that PAs played a role in providing cold tolerance to red spruce (Schaberg et al., 2011). These studies further showed that Put content reverted back to normal homeostatic levels upon amelioration of the stress factor(s). The possible diversion of excess N into the production of N-rich metabolites such as PAs and amino acids (Pro, Arg, GABA, and Glu) as reported for pine stand at HF has also been seen at other N treated sites, e.g., a bog population of three ericaceous shrubs (*Vaccinium myrtilloides, Ledum groenlandicum*, and *Chamaedaphne calyculata*) at Ottawa, Canada (Bubier et al., 2011) indicating that N flux through this part of the pathway works in a coordinated manner. Our studies are consistent with the results of several other groups who have reported coordinated changes in Put and Arg concentrations in the foliage of forest trees growing under environmental stress conditions (Dohmen et al., 1990; Santerre et al., 1990; Ericsson et al., 1993, 1995; Näsholm et al., 1994, 1998).

## Conclusions and future prospects

As much as it is apparent that plants with high PA contents (due to exogenous supply or endogenous production via genetic manipulation) can tolerate short term exposure to a multitude of stress factors, only a handful of studies on the survival and yield (fresh or dry biomass of usable product) in these plants under prolonged stress conditions or repeated exposure to the same stress, have been reported. Most importantly, no viable plant variety has yet been created or selected based upon genetic modification of PAs either via breeding or via transgene expression, which could be evaluated in comparison with other varieties showing similar characteristics. It must also be pointed out that a similar situation exists with respect to a plethora of other genetic manipulation approaches that have been shown to be effective in imparting short-term stress tolerance in various plant species. It is expected that the advanced high through-put techniques of genomics, transcriptomics and proteomics, coupled with better techniques of monitoring the live plants under stress and their metabolic status (the metabolome), would provide a better holistic picture of the consequences of up-regulation or down-regulation of genes likely to be involved in stress tolerance in relation to metabolites like PAs. The ability to identify unique or common regulatory nodes of metabolic pathways, and the cross-talk among the different pathways that are affected by genetic manipulation of PA metabolism, will provide us effective targets to genetically engineer plants that are tolerant to different abiotic stresses. The best-case scenario for such genetic manipulation will be that PA metabolism can be controlled in a transient and cell/tissue/organ specific manner in response to the earliest perception of stress exposure before the stress reaches its peak to cause damage. This would generate plants, which will produce additional PAs to protect themselves from stress only when needed without significant alterations in PA and amino acids metabolism under normal growth conditions.

### Conflict of interest statement

The authors declare that the research was conducted in the absence of any commercial or financial relationships that could be construed as a potential conflict of interest.
